# Congenital Hallux Varus in Children: A Case of Bilateral Presentation

**DOI:** 10.7759/cureus.77844

**Published:** 2025-01-22

**Authors:** Abir Manni, Yacine Zouirech, Badr Rouijel, Sarah Hosni, Tarik El Madhi

**Affiliations:** 1 Department of Pediatric Orthopedic Surgery "B" Children's Hospital of Rabat, Ibn Sina University Hospital Center, Mohammed V University, Faculty of Medicine and Pharmacy of Rabat, Rabat, MAR

**Keywords:** congenital, congenital hallux varus, great toe, medial deviation, surgical treatment

## Abstract

Congenital hallux varus (CHV) is an uncommon deformity of the forefoot, defined by the medial deviation of the great toe at the metatarsophalangeal joint, often accompanied by a wide gap between the first and second toes. This condition is associated with footwear difficulties, pain impacting quality of life, and aesthetic concerns. CHV may occur in isolation or alongside other congenital foot abnormalities, such as polysyndactyly or a longitudinal epiphyseal bracket (LEB). We present the case of a 19-month-old boy with bilateral CHV featuring a shortened first metatarsal and significant functional impairment. Surgical correction resulted in a favorable outcome, emphasizing the importance of early intervention.

## Introduction

Congenital hallux varus (CHV) is an uncommon foot deformity defined by the medial angulation of the great toe at the metatarsophalangeal (MTP) joint [1,2]. In contrast to hallux adductus, which is characterized by the involvement of metatarsus primus varus, CHV is frequently accompanied by an unusually wide gap between the first and second toes, with the severity ranging from mild to extreme cases (up to 90°) [2,3].

Compared to hallux valgus, CHV is much rarer and less frequently congenital [4]. It causes difficulties with footwear, walking, and pain that hampers the quality of life and is cosmetically unacceptable [1,4]. CHV may worsen with growth, particularly when associated with a longitudinal epiphyseal bracket (LEB) [5]. To support normal sensory and motor development, treatment is recommended within the first year of life [6]. Untreated cases may result in persistent deformity and functional impairment due to soft-tissue contractures leading to osseous changes.

Surgical correction is the standard approach, with various techniques described in the literature [1,3,7]. Here, we report a bilateral CHV case in a young child with a shortened first metatarsal successfully treated surgically. Given the uncommon nature of this condition and the limited documentation of CHV in literature, reporting and analyzing such cases is essential to deepen understanding and guide future management strategies.

## Case presentation

**Figure 1:**
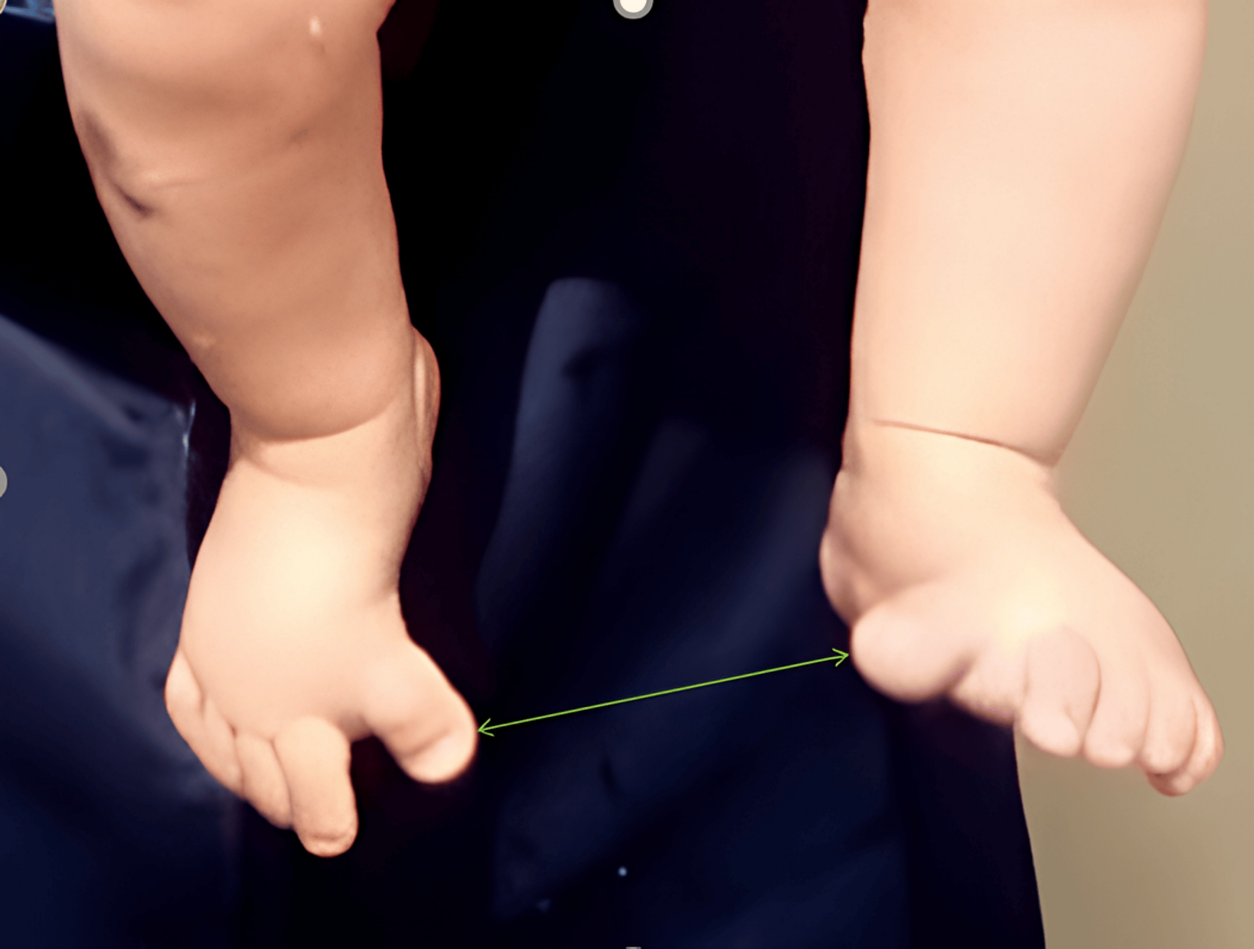
The patient's image at the time of admission Bilateral congenital hallux varus (green arrows) in a 19-month-old boy at the time of admission.

A term-born, 19-month-old boy presented with bilateral CHV to the outpatient department. According to his parents, the deformity was evident at birth and had progressively worsened (Figure 1). The child had no notable obstetric history, with normal prenatal and perinatal development. There were no complications during pregnancy or delivery, and the child exhibited normal physical and mental development. There was no history of trauma, neurological disorders, or familial skeletal abnormalities before presenting to our department.

**Figure 2:**
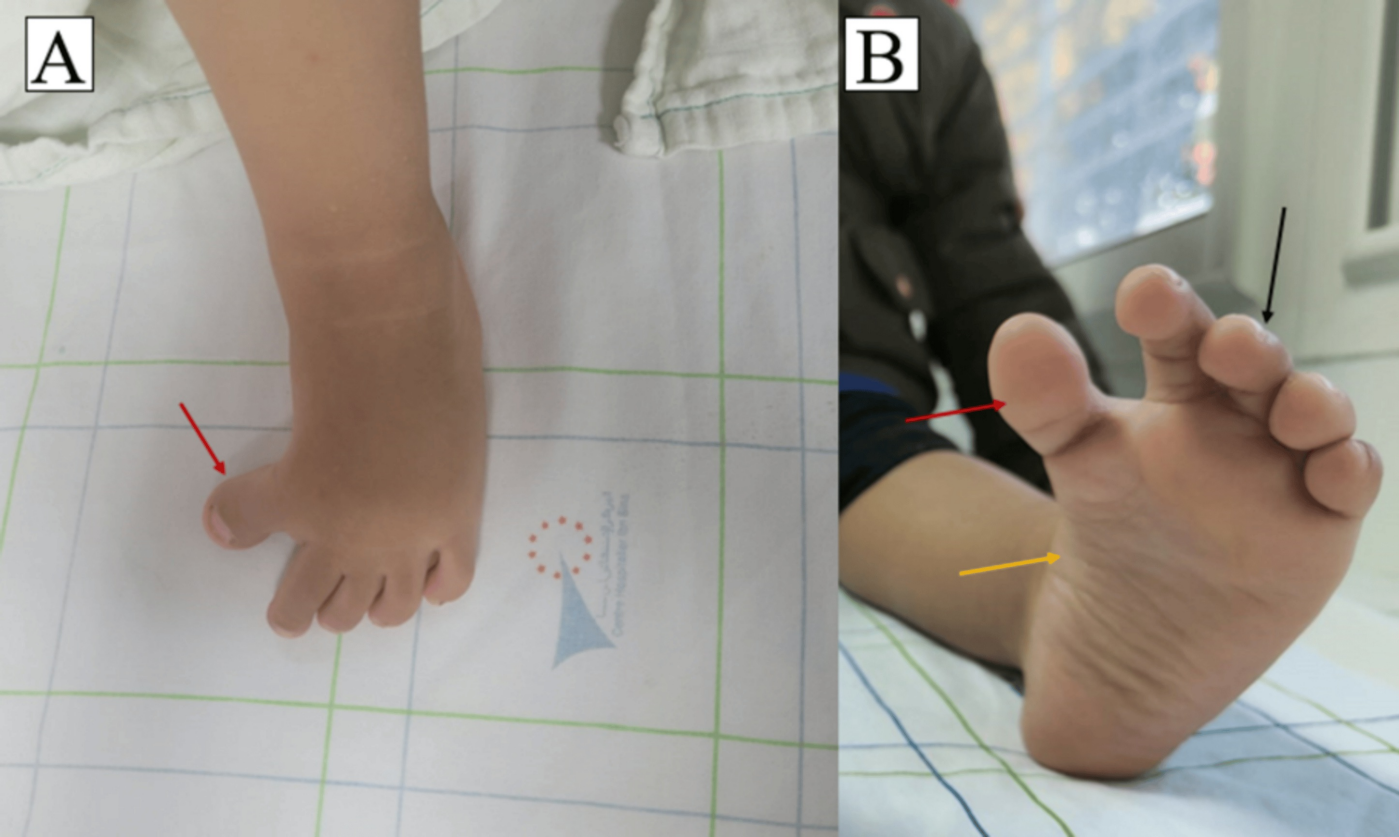
Image of the left foot of the patient with bilateral CHV A. Dorsal view: The red arrow indicates the medial deviation of the great toe (hallux varus). B. Plantar view: Hallux varus (red arrow) is associated with foot inversion and pes cavus (yellow arrow), along with mild clawing of all toes (black arrow). CHV: Congenital hallux varus.

The clinical examination revealed a bilateral varus deformity of the great toes, with angulation exceeding 45° at the metatarsophalangeal (MTP) joint. Attempts to manually realign the hallux to the first metatarsal were unsuccessful, confirming a fixed deformity. Other notable findings included pes cavus, inward foot inversion, and mild clawing of all toes, highlighting the intricate nature of the condition (Figure 2).

**Figure 3:**
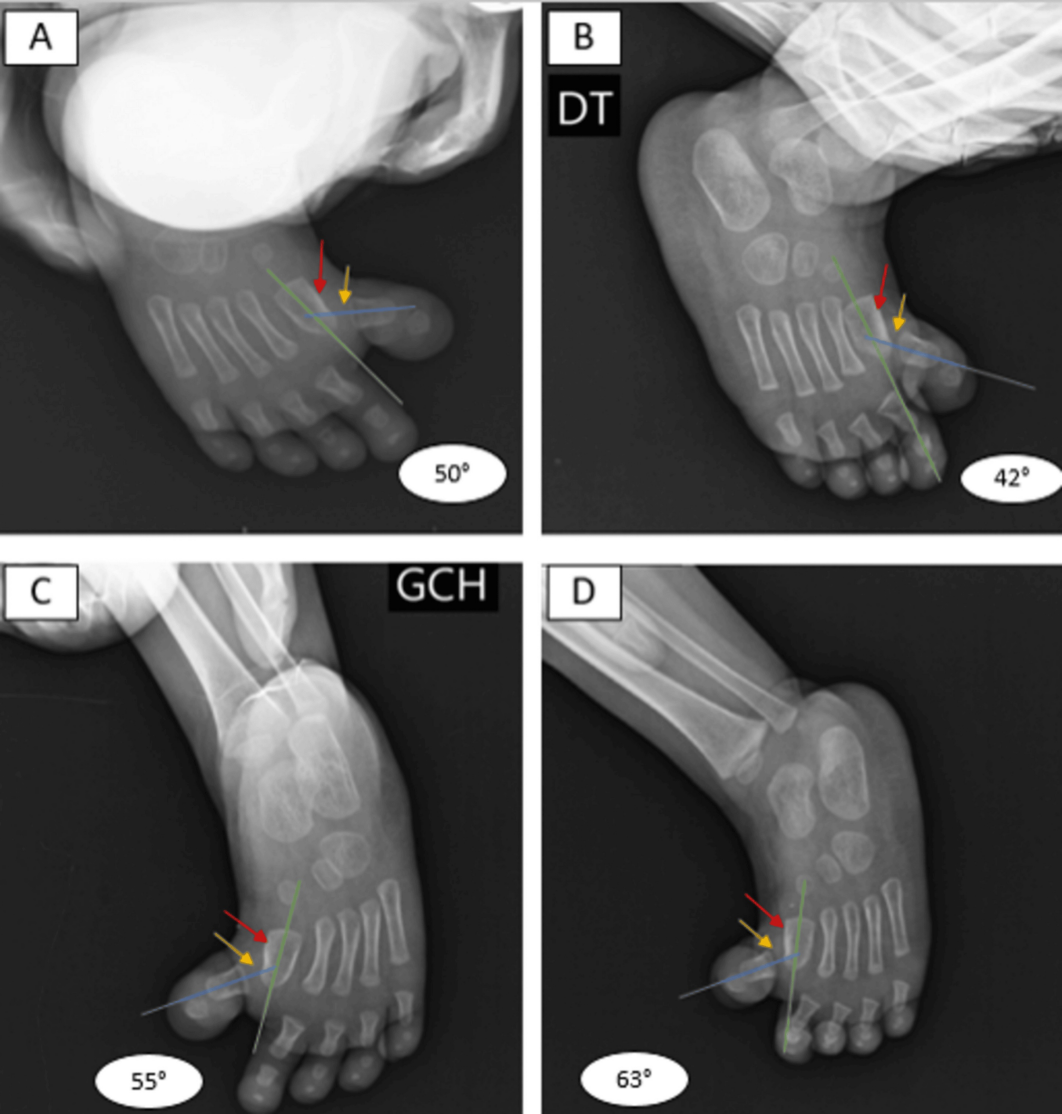
X-ray images of the patient Anteroposterior (A, C) and lateral (B, D) X-ray views of the right (A, B) and left (C, D) feet demonstrating bilateral congenital hallux varus. The deformities were localized at the first metatarsophalangeal (MTP) joint (yellow arrows), with a visibly shortened first metatarsal (red arrows). The angles of deviation were measured as follows: Right foot: 50° in the anteroposterior view (A) and 42° in the lateral view (B). Left foot: 55° in the anteroposterior view (C) and 63° in the lateral view (D). These measurements underscore the severity of the deformity.

Anteroposterior and lateral X-ray views of the left and right feet revealed a medial deviation of the proximal phalanx of the great toe at the first MTP joint, accompanied by a shortening of the first two metatarsals. No associated CHV abnormalities were observed, such as LEB of the first metatarsal, polysyndactyly, or duplication of the first metatarsal. The intermetatarsal angle between the first two metatarsals appeared normal (Figure 3).

The deformities were localized at the first MTP joint (yellow arrows), with a visibly shortened first metatarsal (red arrows). The angles of deviation, calculated between the axis of the first metatarsal (green arrows) and the proximal phalanx of the great toe (blue arrows), were measured as follows: 50° in the anteroposterior view and 42° in the lateral view for the right foot, and 55° in the anteroposterior view and 63° in the lateral view for the left foot. These findings underscore the severity of the deformity (Figure 3).

**Figure 4:**
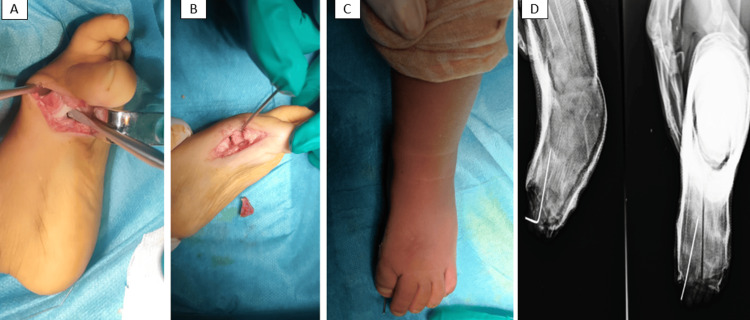
The surgical procedure A: Dorsomedial approach exposing the medial aspect of the foot, with resection of the abductor hallucis muscle (AbH). B: Capsulotomy of the first metatarsophalangeal joint followed by a valgus osteotomy of the first metatarsal. C: Stabilization of the metatarsophalangeal joint using a Kirschner wire. D: Immediate post-operative X-ray showing K-wires in situ.

The surgical correction began with the left foot. A dorsomedial curved incision was performed to access the base of the proximal phalanx and the first metatarsal. The abductor hallucis muscle (AbH) and dense connective tissue on the medial side were removed. Exposure of the medial cortex of the first metatarsal revealed no intraoperative evidence of a LEB. A capsulotomy of the first metatarsophalangeal joint followed, along with a valgus osteotomy to realign the deformity. A bone graft from the anterior superior iliac spine was utilized to stabilize the correction. Finally, syndactylization of the first web space was performed, and the metatarsophalangeal joint was secured with a Kirschner wire (Figure 4).

**Figure 5:**
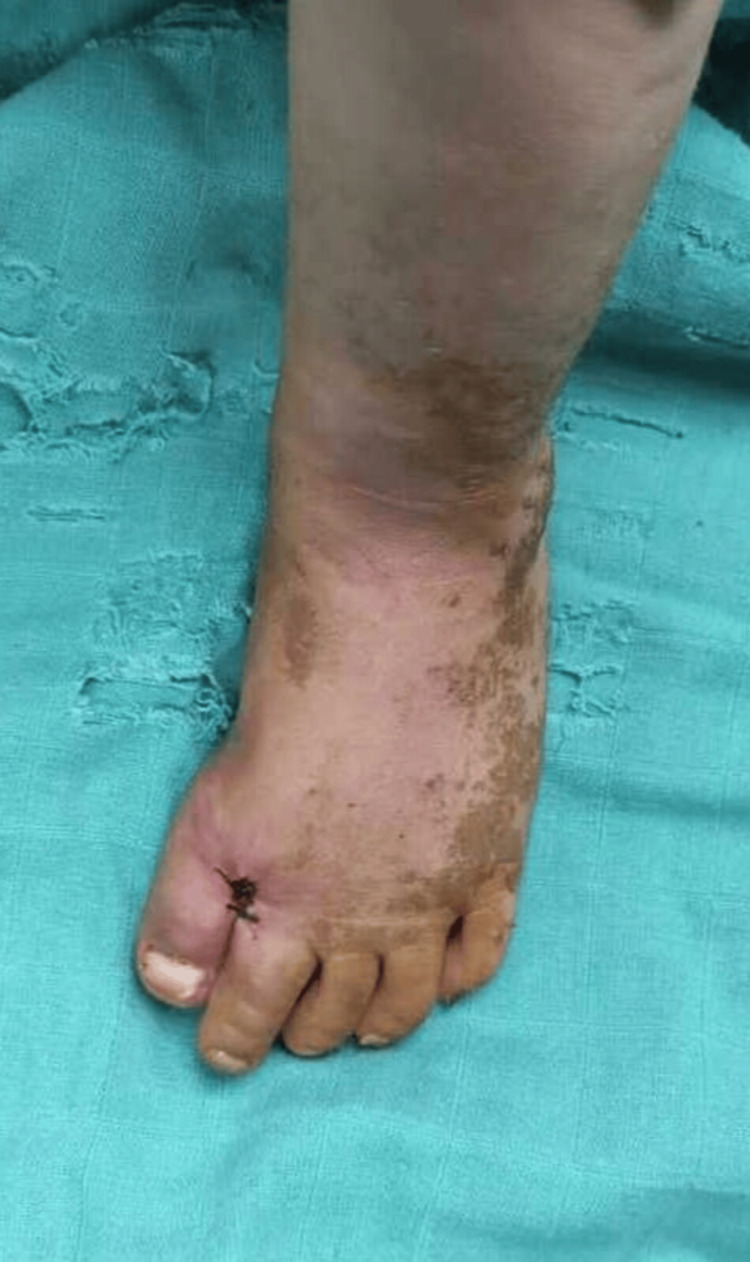
Six weeks post-surgery image Six weeks post-surgery image demonstrating full correction of the varus deformity of the great toe.

Postoperative immobilization with a plaster cast lasted six weeks, after which the cast and wire were removed. Progressive weight-bearing was then initiated (Figure 5).

**Figure 6:**
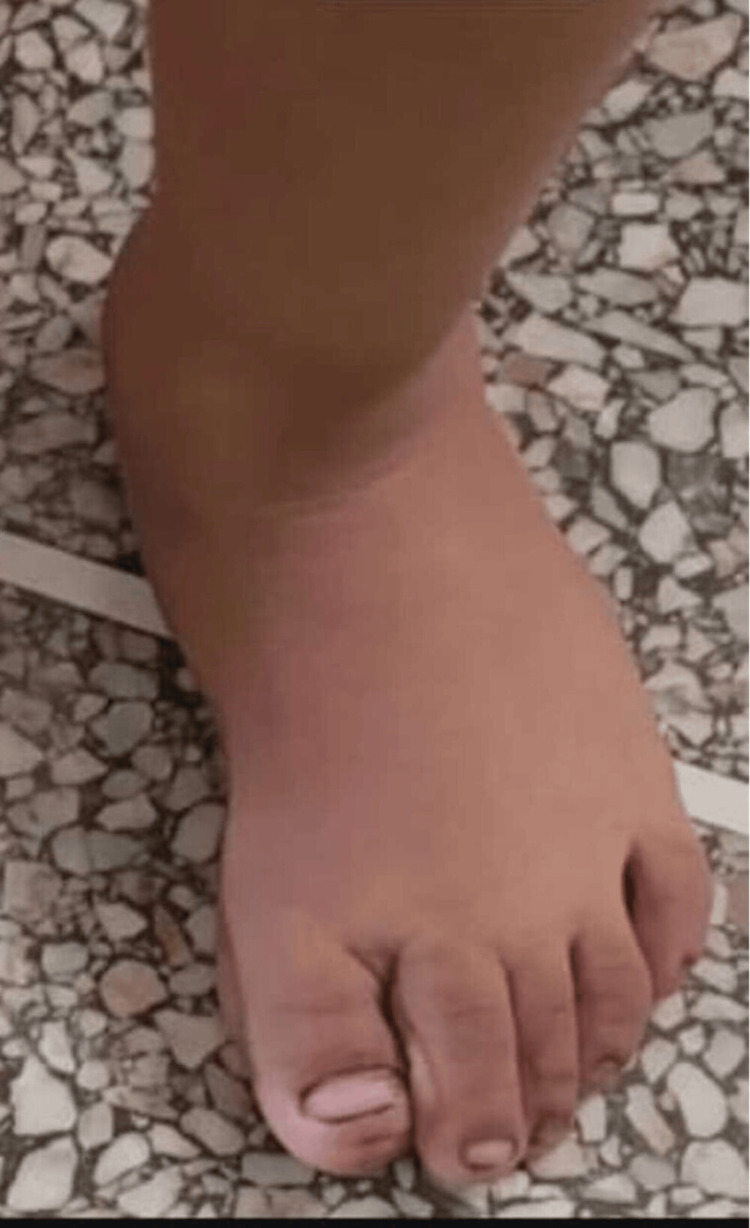
Image after six months of surgery The intervention successfully realigned the great toe, restoring its normal anatomical position and function. No residual deformity or signs of recurrence were observed.

At six months post-surgery, the left foot showed significant improvement in alignment and function. The child demonstrated normal walking patterns with no recurrence of the deformity (Figure 6). Surgical correction of the right foot was planned.

## Discussion

CHV is an uncommon congenital deformity with limited cases reported in the literature, particularly when compared to more prevalent conditions like hallux valgus [4]. CHV's rarity, coupled with the severity of its presentation, often necessitates early intervention to prevent long-term functional impairment and deformity progression [5-6]. As highlighted in the literature, untreated CHV can lead to significant challenges in walking, footwear fitting, and quality of life due to pain and cosmetic concerns [1,4,5].

Surgical intervention remains the treatment of choice, especially in cases where conservative management fails or the deformity is too severe for non-surgical methods. Various surgical techniques have been documented over the past 80 years, reflecting the complexity and variability in the presentation of CHV [1,7,8]. McElvenny and Farmer described early methods involving soft tissue and osseous correction, which have laid the foundation for modern approaches to CHV treatment [1,3]. Techniques range from soft-tissue correction to osteotomy and joint fusion. Tailoring the surgical approach to individual anatomy and functional needs is critical to achieving optimal outcomes and minimizing recurrence risks [5,7].

Soft tissue techniques have been widely adopted by many surgeons, either as standalone procedures or in combination with osseous corrections. These techniques are particularly effective in cases where the first metatarsal is anatomically normal [5]. When supernumerary bones are present, they are generally removed to achieve better anatomical alignment and functional outcomes [5]. Osteotomies are generally reserved for more complex cases, such as those involving a LEB or severe deformities that cannot be fully corrected through soft tissue techniques alone [5].

In some instances, where the first metatarsophalangeal joint has become nonfunctional or painful (often due to neglect or inadequate previous treatment), joint fusion may be considered, though this is relatively rare [9]. In extreme cases where primary surgical interventions fail, amputation of the hallux has been performed as a secondary measure [9]. It is also important to note that first metatarsal lengthening, while sometimes considered for cosmetic purposes, is discouraged because it may disrupt normal foot biomechanics, leading to irregular loading patterns during gait and increasing the risk of further complications [9].

The importance of early detection is increasingly emphasized in recent literature. Aloorkar et al. highlighted the potential for prenatal detection of CHV via antenatal ultrasound, which facilitates early counseling and postnatal planning. Their findings underline the role of multidisciplinary management in optimizing outcomes and addressing potential complications associated with CHV [10]. These considerations underscore the need for a tailored surgical approach, taking into account the specific anatomical and functional requirements of each patient to optimize outcomes and minimize the risk of recurrence or additional deformity.

In the present case, the surgical correction involved a combination of soft tissue release, capsulotomy, and valgus osteotomy, along with the use of a bone graft and temporary fixation with a Kirschner wire. This approach effectively addressed the medial deviation and shortened the first metatarsal, resulting in a favorable outcome.

## Conclusions

CHV is a rare but significant congenital deformity that can severely impact a child's quality of life if left untreated. Early diagnosis and treatment are essential to prevent long-term complications. Surgical correction offers promising results, as demonstrated in this case. Continued follow-up is crucial to monitor for recurrence or late complications, ensuring sustained improvements.
